# Predicting quantum emitter fluctuations with time-series forecasting models

**DOI:** 10.1038/s41598-024-56517-0

**Published:** 2024-03-22

**Authors:** Fereshteh Ramezani, Matthew Strasbourg, Sheikh Parvez, Ravindra Saxena, Deep Jariwala, Nicholas J. Borys, Bradley M. Whitaker

**Affiliations:** 1 Electrical and Computer Engineering Department, Montana State University, Bozeman, USA; 2 Department of Physics, Montana State University, Bozeman, USA; 3 Materials Science Program, Montana State University, Bozeman, USA; 4 Electrical and Systems Engineering, University of Pennsylvania, Philadelphia, USA; 5 Optical Technology Center, Montana State University, Bozeman, USA

**Keywords:** Quantum emitter, Quantum emission, Fluctuations, Forecast, Time-series, LSTM, Prediction, Deep learning, Neural network, Recurrent neural network, Two-dimensional materials, Single photons and quantum effects, Two-dimensional materials, Single photons and quantum effects

## Abstract

2D materials have important fundamental properties allowing for their use in many potential applications, including quantum computing. Various Van der Waals materials, including Tungsten disulfide (WS2), have been employed to showcase attractive device applications such as light emitting diodes, lasers and optical modulators. To maximize the utility and value of integrated quantum photonics, the wavelength, polarization and intensity of the photons from a quantum emission (QE) must be stable. However, random variation of emission energy, caused by the inhomogeneity in the local environment, is a major challenge for all solid-state single photon emitters. In this work, we assess the random nature of the quantum fluctuations, and we present time series forecasting deep learning models to analyse and predict QE fluctuations for the first time. Our trained models can roughly follow the actual trend of the data and, under certain data processing conditions, can predict peaks and dips of the fluctuations. The ability to anticipate these fluctuations will allow physicists to harness quantum fluctuation characteristics to develop novel scientific advances in quantum computing that will greatly benefit quantum technologies.

## Introduction

Quantum phenomena such as superposition and entanglement offer many opportunities to revolutionize secure communication^1^, computation^2^, simulation^3^, and sensing^4^ technologies. Realization of these opportunities necessitates the development of new arsenals of material, devices, and control routines to robustly generate quantum states (qubits) and perform quantum operations (gates). In particular, the field of quantum photonics focuses on using single photons of light as qubits to both transmit quantum information and perform quantum processing operations. Single photons are resilient to decoherence effects, making them ideal quantum information carriers. Thus classical integrated photonic technologies offer an exciting foundation on which to build and deploy wafer-scale quantum photonic systems^5^. Generation of single-photon states is a fundamental requirement for quantum photonics, where the ideal solution has a small footprint to facilitate incorporation in integrated photonic circuitry. Additionally, single-photon states will enable the production of photons at GHz rates, electrical triggers for on-demand photon generation, and indistinguishable photons with identical polarization sates and wavelengths^6^.

Generation of single photons of light with solid-state materials is an attractive solution for quantum light sources for integrated photonics^7^. Unlike solutions that rely on nonlinear optical processes, solid state quantum emitters (QEs) can be triggered on-demand and have the potential for miniaturization for incorporation into integrated photonics. In 2015, solid-state QEs were discovered in the two-dimensional (2D) semiconductor single-layer WSe_2_ (1L-WSe_2_)^8–12^ . While this new class of quantum light sources has many exciting properties, the wavelength (and presumably the polarization) of the photons emitted significantly fluctuates on the timescales of seconds (i.e. spectrally diffuses/drifts/wanders), significantly diminishing their performance in terms of photon indistinguishability^13^. From a materials/devices perspective, increased stability may be achievable by increasing the strain in the system^14^ , tuning the charge density in the material^15^ , and/or modifying the surrounding dielectric environment^16,17^. Additionally, because the QEs in 2D materials are responsive to external stimuli^18^, the emitters have the potential to be actively monitored and stabilized with a feedback loop during operation. Just recently, a revolutionary concept of a smart quantum camera that leverages artificial intelligence to discern statistical fluctuations of unknown mixtures of light sources has been introduced^19^. The incorporation of a universal quantum model and artificial neural networks not only surpasses existing superresolution limitations but also presents new opportunities in microscopy, remote sensing, and astronomy. Equally noteworthy is the exploration of shaping light beams with varied photon statistics, that offers a remarkable degree of control over spatially varying photon fluctuations^20^.

Here, we present an initial exploration of the ability for an neural network-based machine learning algorithm to predict the fluctuation of QEs in 2D materials based on the immediate history of its emission. Such a predictive algorithm could be used to improve photon indistinguishability of an advanced QE device where the emission wavelength is monitored and external stimuli is applied to prevent predicted fluctuations. Our results show that a neural network that is rudimentary trained on the intensity fluctuations of discrete wavelength bins may be able to forecast future fluctuations. The work sets the stage for more sophisticated training strategies that take into account both emitter intensity and peak emission wavelengths.

### Fluctuations of QEs in 1L-WS_2_

**Figure 1:**
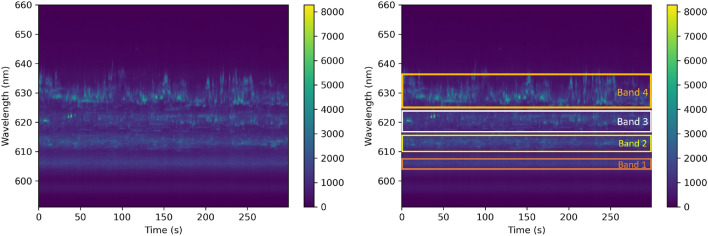
Fluctuations in intensity and energy for a nanobubble QE in single-layer WS_2_ on an Au surface. The figure on the left is shows the number of photons emitted by the device as a function of time and photon wavelength. The figure on the right shows the same data, but also indicates four different wavelength bands where quantum emissions occur.

Figure 1 shows representative deleterious fluctuations of QEs from randomly occuring nanobubbles^21^ in single-layer WS_2_ that is deposited on a gold surface with gold-assited exfoliation techniques^22,23^. The QEs are identified has narrowband emission lines that are superimposed on a weaker background of broader, excitonic states. The time evolution of the QEs is measured by repeatedly acquiring individual photoluminescence spectra every 2 seconds over a total duration of several minutes. The time series dataset is constructed by appending individual spectra acquired in nearly continuous succession. The temporal delay between each acquisition is less than 500 ms. See Methods for additional details of the experimental apparatus. Inspection of the time evolution reveals that the center wavelengths and the emission intensities of individual states change on the timescale of seconds. These fluctuations are likely caused by the local environment surrounding the emitter, including nearby metallic and dielectric structures. QEs are identified in four wavelength bands that are centered at 606 nm, 613 nm, 621 nm, and 629 nm. Large-area encapsulation of the devices with thin layers of hBN provides potential means of mitigating these fluctuations. However, they are not an absolute solution to eliminate them completely. To address further stability, QEs that are dynamically tunable can be used alongside a feedback system that intermittently samples QLED emission and applies corrective stimuli. Machine learning time-series forecasting models have the potential to predict QE fluctuations and provide physicists with essential information to apply corrective stimuli to stabilize their emission wavelength and intensity in real time.

### Time-series forecasting

Time-series forecasting is an important applied machine learning technique that includes developing models to analyse (describe and predict) sequences of data collected during time intervals. These models are widely used in different areas such as finance^24^, sales forecasting^25^, climate time series^26^, pore-water pressure^27^, and medicine^28^, to name a few. In time-series forecasting models, future values of a target $y_{i,t}$ for a given entity i at time t is predicted. The entity displays a logical grouping of temporal information. The most straightforward models which can predict the one-step-ahead can be represented as below:

$$\begin{aligned} \hat{y}_{i,t+1}=f(y_{i,t-k:t}, x_{i,t-k:t}, s_i) \end{aligned}$$

In this equation, $\hat{y}_{i,t+1}$ represents the model forecast, $y_{i,t-k:t}$ represent observations of the target for the previous *k* samples, $x_{i,t-k:t}$ represents observations of the input over the previous *k* samples, $s_i$ is static meta data linked to the entity, and $f(\cdot )$ is the prediction model function which is learned by the model^29^.

#### Recurrent neural networks

**Figure 2:**
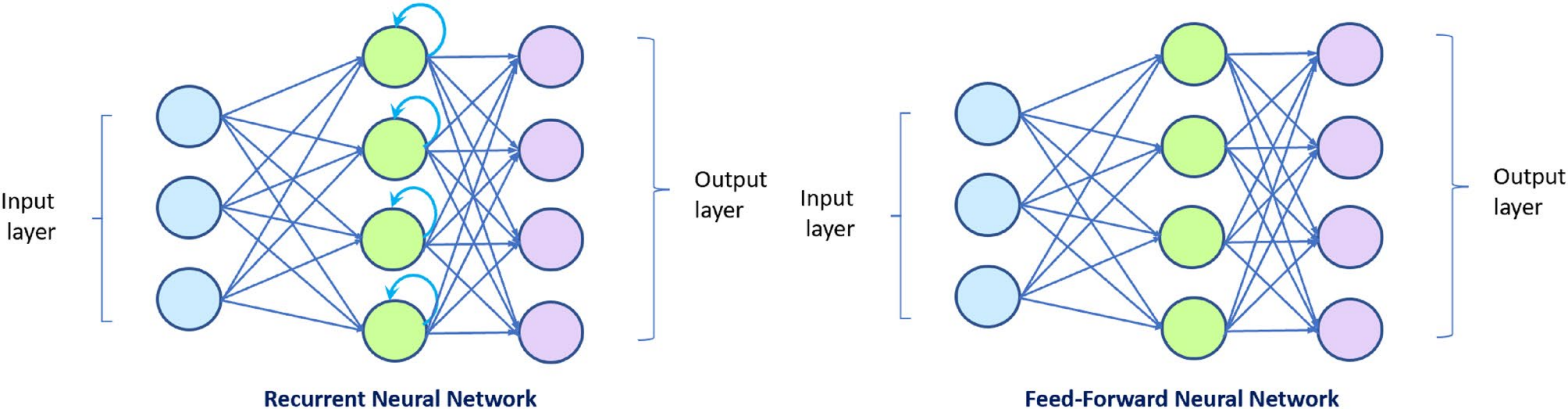
Recurrent neural network and feed-forward neural network architectures. The figure on the left shows a recurrent neural network with recurrent connections on hidden states which do the back propagation, whereas the figure on the right shoes the feed-forward architecture of neural networks that have no recurrent.

Deep learning algorithms have been developed and frequently used to extract information from many types of data during the last several years^30,31,31^. Recurrent neural networks (RNNs) are types of deep learning networks that can be used to analyse temporal information due to their specific architecture feedback loops which is their memory. They take information from prior inputs and update the current input and output^30^ whereas in traditional neural networks, inputs and outputs are independent from each other. Figure 2 illustrates the difference between a traditional feed-forward neural network and a recurrent neural network.

**Figure 3:**
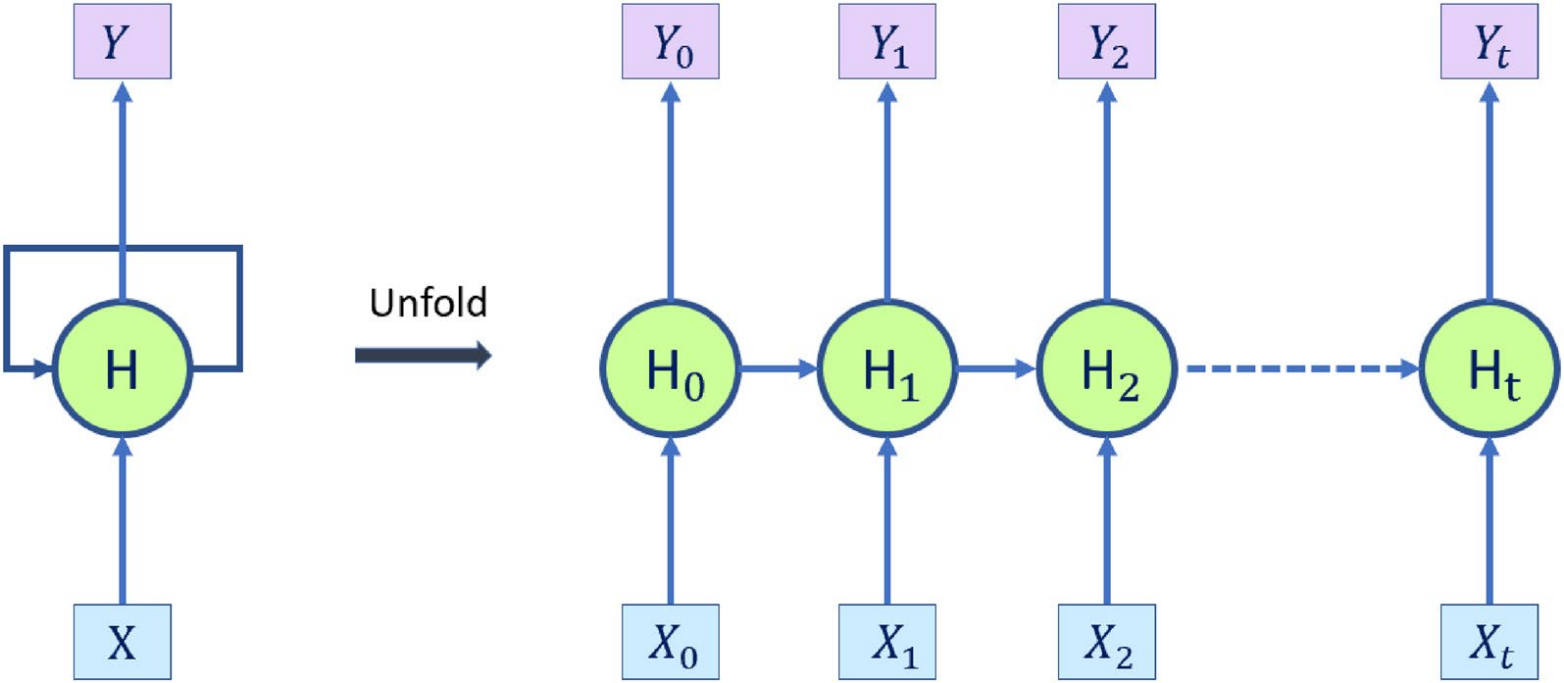
Unfolded sequential architecture in an RNN. $X_t$ is the input at time step t, $Y_t$ is the output at time step t and $H_t$ is the hidden state at time t. The repeating modules are designed to act as a collective memory, sharing parameters across various time steps to store important data from earlier processing stages.

To be able to process the sequence data, RNNs have repeating modules arranged in chains (Fig. 3), where they can share the parameters across different time steps with the intention of employing these modules as a memory to store crucial data from earlier processing steps. The capacity of recurrent neural networks make them suitable for many applications such as natural language processing and language modeling^32,33^, speech recognition^34,35^ and emotion recognition in videos^36^.

Over time, various kinds of RNNs have been developed and applied to temporal forecasting problems with strong results^37–40^. Recurrent connections can enhance neural network performance by taking use of their capacity for comprehending sequential dependencies. However, the techniques used for training RNNs might significantly restrict the memory generated from the recurrent connections. Older variants of RNNs suffer from exploding or vanishing gradients during the training phase, causing the network to fail to learn long term sequential dependencies in data^41^.

**Figure 4:**
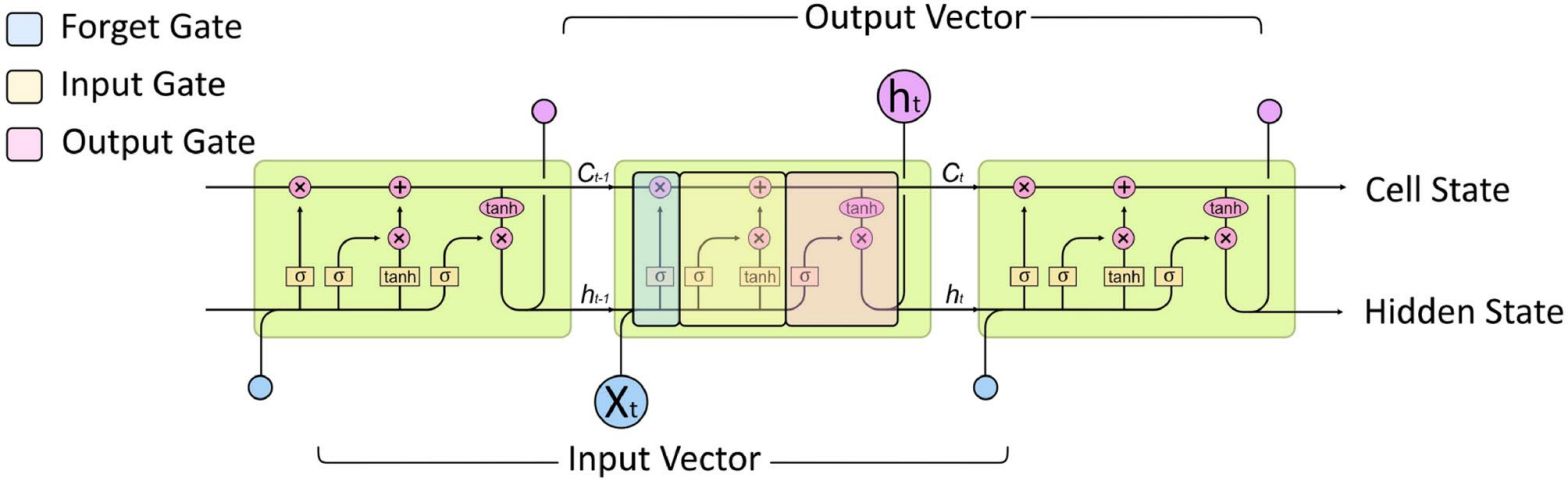
Memory cells structure in LSTM^44^. These cells are comprised of three gates: an update or input gate, a forget gate, and an output gate. Together, these gates facilitate the learning of long-term dependencies. The input gate and output gate determine what information to update and transmit to the next cell, respectively. On the other hand, the forget gate decides which information is least relevant and should be discarded.

*Explanation of LSTM networks* Long short-term memory (LSTM) is the most popular method and model to address the insufficient memory of RRNs and tackling the problem of exploding and vanishing gradients. LSTM was introduced in 1997 for the first time to address the problem of long lasting learning to store information over extended time intervals by recurrent backpropagation^42^. In LSTM, instead of sigmoid or tanh activation functions, there are memory cells with inputs and outputs which are controlled by gates^42,43^. In other words, LSTM is a modified version of RNN made of repeating modules called memory cells. These cells consist of three gates: an update or input gate, a forget gate, and an output gate. These gates work together handle learning long term dependencies. The input gate and output gate decide what information to update and pass on to the next cell respectively, while the the forget gate decides the least relative information to through away. This structure is shown in Fig. 4.

*LSTM applications* LSTM neural networks can be applied to various tasks including prediction, pattern classification, recognition and analysis. Since LSTM is able to process sequential data, it is an effective tool in many different domains such as statistics, linguistics, medicine, transportation, computer science and more^45^. As instance, in^46^ LSTM based models are used in natural language processing (NLP) for sequence tagging. In another work, Xue et al.^47^ have applied LSTM to pedestrian trajectory prediction. They have proposed a network based on LSTM which is able to consider both influence of social neighbourhood and scene layouts in pedestrian trajectory prediction. Precipitation nowcasting is a crucial and challenging weather forecasting problem, which the goal is to predict the future rainfall intensity in a local region over a relatively short period of time. Shi et al.^48^ have used LSTM networks to build an end-to-end trainable model for the precipitation nowcasting problem. These instances are just a few of numerous applications of time-series forecasting models that are under research and applied in forecasting problems. Achieving good and strong results in recent work, was a motivation for us to apply time-series forecasting models to the QE fluctuation in intensity and energy problem.

#### Correlation and autocorrelation

Various physical phenomenon can cause variables within a dataset to be relevant or fluctuate in relation to each other. Finding and quantifying how reliant the variables in a data set are on one another or on themselves is crucial in many machine learning algorithms, including time-series forecasting problems to ensure the possibility of time-series predictions. Correlation is a statistical measure that indicates this relationship. Correlation can be either positive, negative or zero. A positive correlation indicates that variables change in the same direction. A negative correlation indicates that the variables change in opposite directions, meaning that while one increases, the other decreases. A zero correlation indicates that the variables have no relationship with each other. Therefore, if a signal has zero autocorrelation after a certain number of time lags, then it is theoretically impossible to predict the future states of that signal using only past measurements of that signal. The nonzero cross-correlation and autocorrelation indicates that it should be possible to perform meaningful predictions in the time-series data. The correlation coefficient is a unit-free statistical measure in the range of -1 and +1 that reports the correlation between variables. One of the most popular coefficient is Pearson’s coefficient also known as the product moment correlation coefficient. It is the ratio between the covariance of two variables and the product of their standard deviations. Given a pair of variables (X,Y), the formula for the coefficient represented by $\rho$ is defined as below:

$$\begin{aligned} \rho = \frac{\text {cov}(X,Y)}{\sigma _x \sigma _y}. \end{aligned}$$

In this formulation, cov is the covariance, $\sigma _x$ is the standard deviation of *X* and $\sigma _y$ is the standard deviation of *Y*. For a sample and given paired data of *X* and *Y*, the Pearson’s coefficient is commonly represented by *r* and can be expressed in terms of mean and expectation as below:

$$\begin{aligned} r = \frac{{}\sum _{i=1}^{n} (x_i - \overline{x})(y_i - \overline{y})}{\sqrt{\sum _{i=1}^{n} (x_i - \overline{x})^2(y_i - \overline{y})^2}}, \end{aligned}$$

where *n* is the sample size, $x_i$ and $y_i$ are the sample points indexed with *i*, and $\overline{x}$ and $\overline{y}$ are the sample means.

In time series problems, analyzing the correlation between a series and lagged versions of itself can provide predictive information. The correlation coefficient between a time series and its lagged versions over time intervals is called autocorrelation. In other words, autocorrelation determines the degree of similarity between a series and its lagged counterparts. Autocorrelation analysis is a popular tool for determining the degree of randomness in a data set. By measuring and analysing the autocorrelations for data values at various time lags, this randomness is determined. If the nature of the data over time is random, such auto correlations ought to be close to zero for all time-lag separations. If the autocorrelation at one or more lag samples are significantly non-zero, then the data is not random^49^.

**Figure 5:**
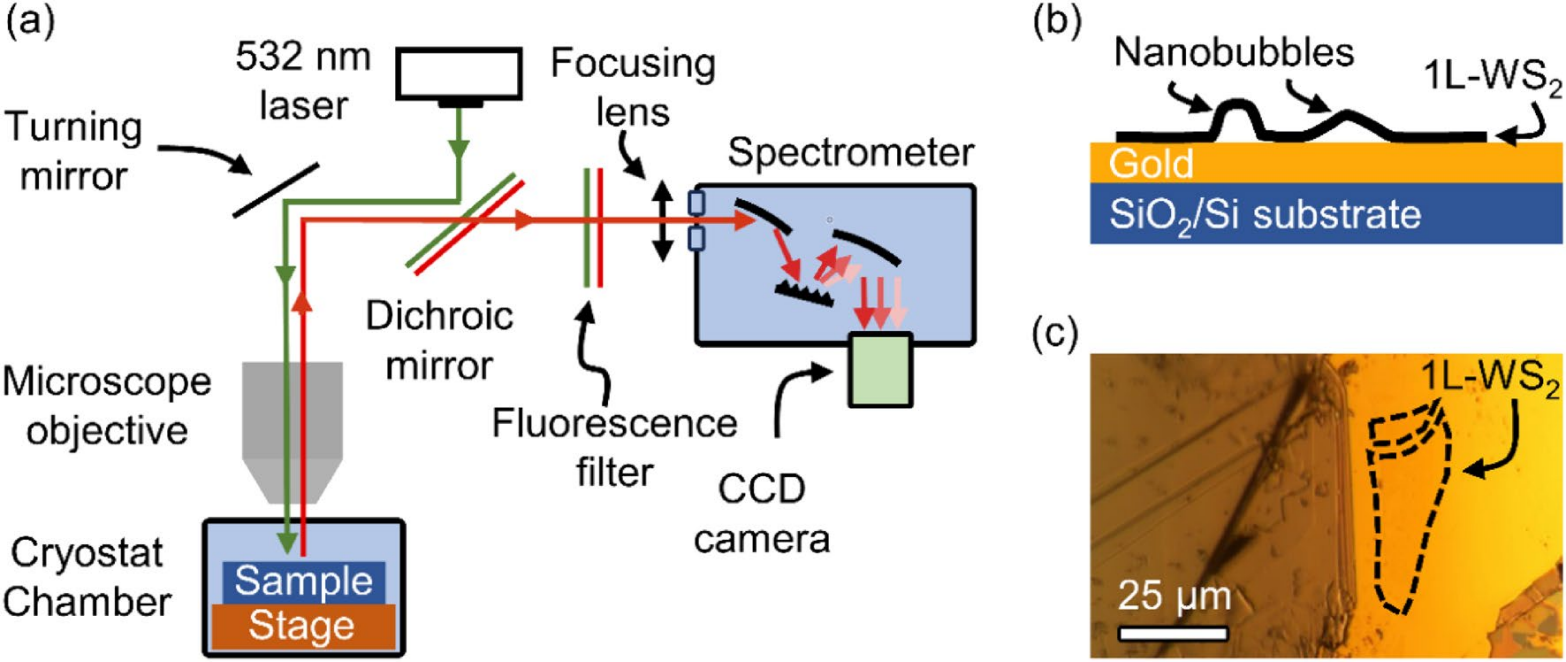
The experimental setup used to acquire the time-series datasets. (**a**) A schematic of the optical microscope used to measure the photoluminescence at a sample temperature of 4 K. A 532 nm continuous wave diode laser is used to excite the sample and a 532 nm long pass dichroic mirror and fluorescence filter is used to reject the laser light before focusing the photoluminescence into a grating spectrometer. (**b**) A schematic of the cross-section of the sample which consists of 1L-WS_2_ nanobubbles on a gold substrate. (**c**) An optical micrograph of the sample. The regions that contain the 1L-WS_2_ with nanobubble are outlined by the dashed lines.

**Figure 6:**
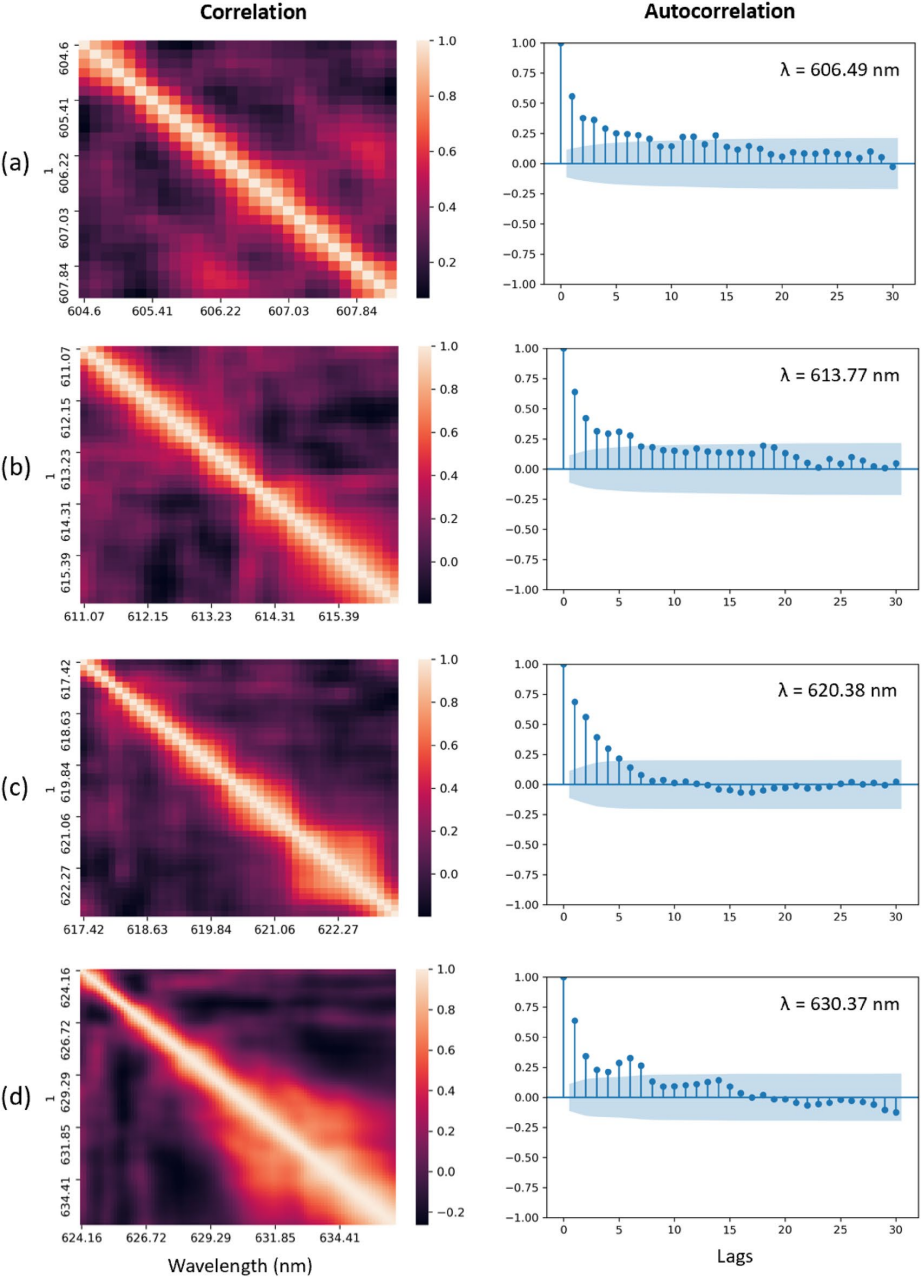
Correlation matrix and autocorrelation plot for 4 bands and their centers for 30 lags. (**a**), image on the left represents the correlation matrix for wavelengths withing band 1 and the image on the right represents the autocorrelation lambda = 606.49 nm. (**b**), image on the left represents the correlation matrix for wavelengths withing band 2 and the image on the right represents the autocorrelation for lambda = 613.77 nm. (**c**), image on the left represents the correlation matrix for wavelengths withing band 3 and the image on the right represents the autocorrelation for the lambda = 620.38 nm. (**d**), image on the left represents the correlation matrix for wavelengths withing band 4 and the image on the right represents the autocorrelation for lambda = 630.37 nm.

## Methodology

### Data acquisition

Figure 5a shows a schematic of the optical microscope used to measure the luminescence of single 1L-WS_2_ nanobubbles. The optical setup includes a standard confocal photoluminescence microscope built around a closed-cycle liquid helium cryostat with optical access (Montana Instruments)^50–52^. The nanobubble samples consist of 1L-WS_2_ that is transferred on top of a smooth gold surface, as shown schematically in Fig. 5b and by the optical micrograph in Fig. 5c. Nanobubbles form at the 1L-WS_2_/gold interface due to the coalescence of contamination that was trapped in the interface during fabrication^21^. The sample is cooled to 4 K to slow the ionization of the QE states due to heat and a laser beam (wavelength = 532 nm, continuous wave) is focused onto an area (1 × 1 µm^2^) that contains narrowband emitters with energies less than the exciton state of 1L-WS_2_. The power of the excitation laser is adjusted to be below the saturation threshold of the emitters, which is common practice for achieving high emitter purity and for spectroscopic characterization of quantum emitters in 2D semiconductors^53,54^. The photoluminescence from the sample is collected and spectrally isolated from the reflected laser light with a series of thin-film interference filters (532 nm long pass filters). The fluorescence is then spectrally dispersed with a Czerny–Turner optical spectrometer (Princeton Instruments) and measured with a cooled scientific CCD camera (Princeton Instruments). The continuous series of 90 spectra were acquired from an emissive region of the sample with integration times of 2 s, spanning a duration of about 180 s.

### Preprocessing

In order to learn historical values and patterns to forecast the future, there needs to be a window of time that is fed to the algorithm, so it can make predictions based on the previous values. For this, we transformed the data into input and output pairs where the observations at 5 previous time steps (a window size of 10 s) are used as inputs to make predictions for one step into the future (2 s). To later train and evaluate the forecast models, we divided this dataset into three subsets: a training set, a validation set and a test set. The training set consists of the first 80% of the time sequence, the validation data consists of the next 10%, and the test data consists of the last 10% of the time sequence data. This ensures that the time-series properties of the data are consistent within a particular subset of data, and that the test data has truly never been seen by the classifier before.

The performance of machine learning models depends on the quality of the data. There are various preprocessing techniques which convert the data into useful information to the models. Among various techniques, data normalization is an approach which entails scaling or transforming the data to ensure that each feature contributes equally and avoid bias towards a specific range of values in the feature data. As part of the proprocessing of our algorithm, we first checked the dataset for negative photons counts and zeroed them out as they are produced due to noise and/or calibration error. Then we normalized the data based on the statistical mean and standard deviation of the training set. Each value of the data (including those in the validation and test sets) is converted as follows, where $X_{new}$ is the normalized value, $M$ is the statistical mean of the training set and $\sigma$ is the standard deviation of the training set:

$$\begin{aligned} X_{new} = \frac{X - M}{\sigma }. \end{aligned}$$

### Experiment and model

To forecast the photon intensities, we designed two different scenarios:

1. Prediction considering all measured wavelengths.
2. Prediction considering 4 different bands including the most correlated wavelengths.

For the prediction models, we developed a multivariate LSTM model and a shifted forecast that considers the current measurement as a prediction for the next step. Using a shift in the data is the simplest method of forecasting, which is a basic technique that assumes the future value of a variable will be similar to its most recent past value. This method is often referred to as the naive forecast. It provides a quick and straightforward baseline for comparison with more sophisticated forecasting methods. As there are more than one variable in the dataset, a multivariate time-series forecast can be used. This will consist of multiple variables, each of which is dependent on other variables to some extent, in addition to its own historical values. For the first scenario, we developed a model with inputs of 5 time steps (10 s) over all the wavelengths regardless of their correlations, and then we did predictions for the values one-single step into the future. For the second scenario, we divided the wavelengths into the 4 bands that include the most correlated wavelengths (see Fig.1). Then we trained the model on each band individually considering the same time steps as the first experiment and predicted one-step in the feature.

In our research we used various open libraries in Python, such as Tensorflow, Matplotlib, Pandas, Numpy for preprocessing, training and visualisation purposes. Our LSTM model consists of one LSTM layer followed by two fully connected layers to predict the feature values. We employed a sequence length of 10 s as inputs, and predicted 2 s in the future. It took about 15 mins to train the algorithm on all the measured wavelengths (first scenario) using an Intel(R) Core(TM) i9-8950HK CPU @ 2.90GHz, 2904 Mhz, 6 Core(s), 12 Logical Processor. All other models took less time to train. The LSTM models were trained using Adam optimizer with a learning rate of 0.0001 and a mean squared error as the loss function. Furthermore, during the training, we employed callbacks to prevent the overfitting of the network and saving the best performance results based on the error obtained on the validation set.

### Evaluation

To evaluate the models, we used root mean square error (RMSE) as our cost function which is commonly used in forecasting problems^55–57^. Considering *n* as the number of samples, $y_i$ the actual value and $\hat{y_i}$ predicted value, RMSE is defined as:

$$\begin{aligned} \text {RMSE} = \sqrt{\frac{1}{n}\Sigma _{i=1}^{n}{\Big ({y_i -\hat{y_i}}{}\Big )^2}}. \end{aligned}$$

For each of the experiments and models, we report the RMSE. We also present plots of the actual intensity values and predicted values, as well as the differences between these two.

## Results

### Correlation

To divide the data to bands with the highest correlations, we measured the correlation matrix and picked the wavelengths with the highest correlation coefficient using Pearson’s algorithm. This resulted in 4 bands with different numbers of wavelengths, as described in Table 1. The correlation coefficient matrices are shown in Fig. 6. Before starting to train the time-series forecasting models, we assessed the randomness of the data to ensure the problem is predictable. We measured the autocorrelation for the dataset for 30 lags (60 s) and plotted the center of each band for illustration purpose in Fig. 6. The blue shaded area is the confidence interval and represents the significance threshold. This indicates that autocorrelations inside the blue area are statistically insignificant, and anything outside of it is statistically significant. It is visible that, for each of the wavelengths, there are several autocorrelations that are significantly bigger than zero, therefore the time series dataset is not completely random.

### Prediction

**Figure 7:**
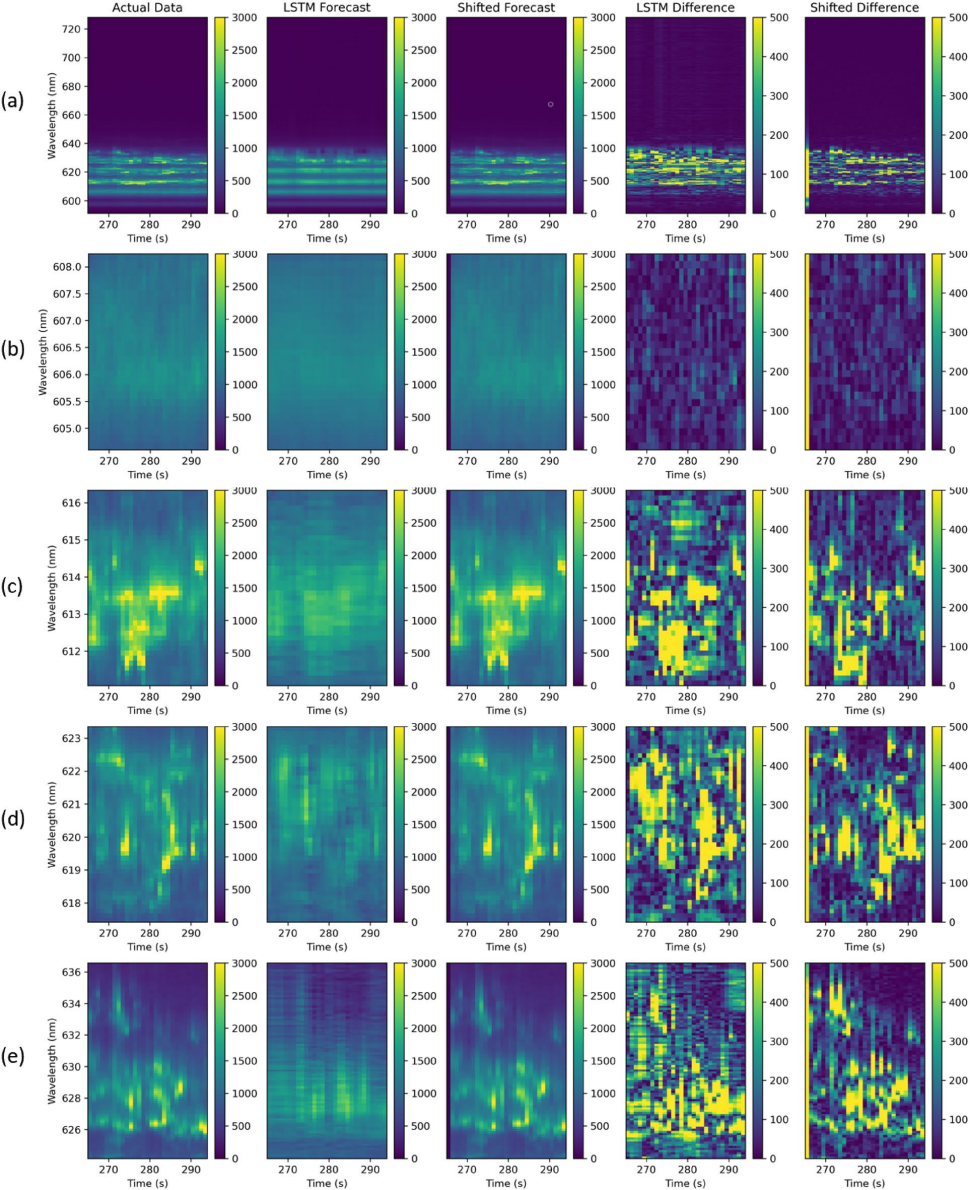
Prediction results using LSTM and shifted forecasts. Columns represent the actual image, LSTM forecast, shifted forecast, difference between the actual image and LSTM forecast, and difference between the actual image and shifted forecast, respectively. Row (**a**) corresponds to the full image where all the wavelengths within the dataset are considered. Row (**b**–**e**) correspond to the band 1, band 2, band 3, and band 4, respectively.

After checking the non-randomness nature of the data and developing the prediction models, we applied the trained models to the test sets and measured the RMSE. The measurement results are reported in Table 1. For each of the bands, the RMSE is calculated twice for LSTM prediction; once considering the model trained on full image (all the wavelengths) and another time considering the model trained only on the subset of wavelengths within the band. For almost all the experiments, the LSTM model does a better job than the shifted forecast; significantly for band 1 and band 2. The RMSE values measured for LSTM forecast show that higher correlations help the models to learn the historical data better, and therefore they lead to improvements in prediction results. In Fig. 7, we have displayed the prediction images using both LSTM forecast and the shifted forecast. For comparison of both models, we have shown the differences between the actual image and predicted image.

**Table 1:** Root mean square error using LSTM and shifted forecast.

	Wavelength	Number of	Root mean square error		
	Range (nm)	Bins	LSTM (band)	LSTM (full Image)	Shift
Full image	591.12–728.15	1023	79.12	79.12	79.40
Band 1	604.06–608.24	28	76.26	103.42	225.74
Band 2	611.07–616.34	40	313.60	336.80	387.19
Band 3	617.42–623.35	45	363.44	381.88	368.40
Band 4	624.16–636.57	93	305.54	326.70	296.05

**Figure 8:**
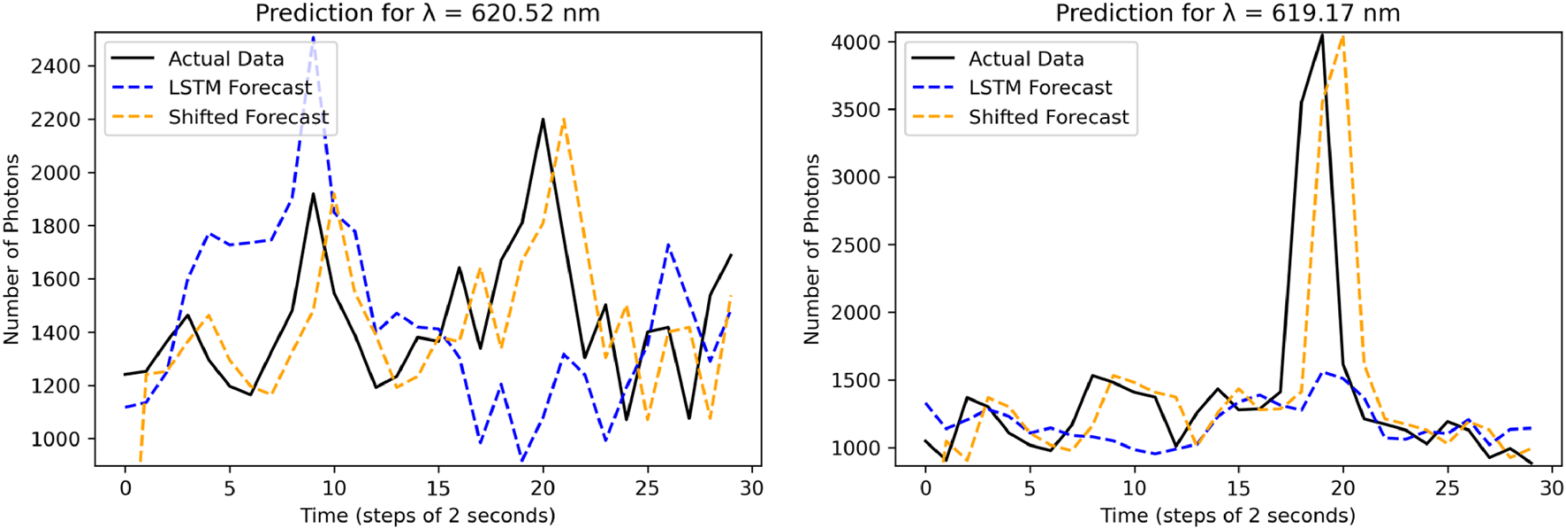
Single step prediction for wavelength 620.52 nm and 619.17 nm using LSTM forecast and Shifted forecast. For 620.52 nm, LSTM and shifted forecast RMSE are 420.37 and 346.7. For 619.17 nm, LSTM and shifted forecast RMSE are 648.80 and 654.57 respectively.

Figure 8 shows the forecast results for two wavelengths belonging to band 3. For wavelength 620.52 nm, the LSTM model was able to fairly predict and follow the actual trend. For example it was able to predict several peaks and troughs, at the times 9–12 s and 17–18 s. For the wavelength of 619.17 nm, the LSTM model was able to detect the highest peak even though the predicted value is far from the actual data in terms of intensity. In both plots, there are many peaks and troughs that were not predicted by the LSTM model. Also note that the shifted forecast, by definition, identifies all peaks and troughs, but at a delayed time. The delay in QE prediction makes a simple shifted forecast infeasible to incorporate into a feedback control system.

As shown in Table 1, when the algorithm is applied to a narrow band (Band 1, 4.18 nm wide) the RMSE improves from 225.74 to 76.26. However, when applied to the wider bands, (Band 2 = 5.27 nm; Band 3 = 5.93 nm; Band 4 = 12.41 nm) and on the full image (137.03 nm) the performance between the LSTM method and the shifted forecasting method is comparable. Based on these results, as well as the correlation matrices presented in Fig. 5, it seems that the information required for effectively predicting quantum fluctuations lies in a fairly small wavelength band. Future work will investigate this hypothesis by creating and analyzing datasets of narrowband quantum fluctuations.

## Discussion

To the best of our of knowledge, the development of high-performance models that can forecast the fluctuations of quantum emitters (and other nanoscale light sources) has not been studied prior to this work. Being able to forecast these fluctuations would provide deep insight into their origins and enable scientists and engineers to pursue predictive stabilization of nanoscale solid-state light sources. In this work, we developed a LSTM-based models towards the prediction of QE fluctuations over time for emitters in 1L-WS_2_ on gold. These emitters show dramatic fluctuations and providing and extreme test of the ability for neural network models to identify trends in the fluctuations. Despite the challenge of the task, our trained model was able to predict the general trend of a quantum emitter better than a simple shifted forecast, as measured by improved RMSE. To some extent, the model was able to predict the peaks and troughs of the fluctuations, though this behavior was inconsistent. We also showed that the fluctuations are of non-random nature, as measured by correlation and autocorrelation. Building on this initial step, our future work will focus on increasing the size and durations of the datasets on which the neural network is trained and explicitly training the neural network on wavelength and intensity fluctuations (as opposed to just the wavelength bands used here). We also plan to characterize the neural network as a function of brightness by increasing the size of our dataset which we think would be an interesting study to further explore this context. Additionally future work will investigate the relationship between model performance and the wavelength bandwidth. These next steps will also lead to developing multi-step, multivariate time-series forecasting which could predict multiple steps into the future.

## Data Availability

To help the other researchers and scientists explore improved methods for solving quantum emitter fluctuations, the data and ode are available at https://doi.org/10.5281/zenodo.8320019. Additional questions about the data should be directed to author B. Whitaker at bradley.whitaker1@montana.edu.
